# A Mathematical Approach on the Limits of ceRNA Hypothesis Through an Ordinary Differential Equations (ODE) Model of mRNA-microRNA Interactions

**DOI:** 10.3390/ncrna12040022

**Published:** 2026-06-29

**Authors:** Paul Flondor, Mircea Olteanu, Radu Stefan, Corina Elena Minciuna, Catalin Vasilescu

**Affiliations:** 1 Faculty of Automatic Control and Computer Science, National University of Science and Technology POLITEHNICA Bucharest, 313 Splaiul Independentei, 060042 Bucharest, Romaniamircea.olteanu@upb.ro (M.O.); radu.stefan@upb.ro (R.S.); 2 Department General Surgery I, Fundeni Clinical Institute, 258 Sos. Fundeni, 022328 Bucharest, Romania; corina.minciuna@umfcd.ro; 3 General Surgery Department, University of Medicine and Pharmacy Carol Davila, Blvd. Eroii Sanitari 8, 050474 Bucharest, Romania

**Keywords:** ODE model, equilibria, stability, protein production, homeostasis, microRNA, non-coding RNA, ceRNA, 34D23, 37B25, 7C10, 37C70, 37C75

## Abstract

Background: MicroRNAs (miRNAs) are small, non-coding RNA molecules that regulate gene expression post-transcriptionally by binding to target messenger RNAs (mRNAs) and suppressing their expression. Competing endogenous RNAs (ceRNAs), including mRNAs and circular RNAs (circRNAs), modulate miRNA availability through competitive binding, forming regulatory networks that fine-tune gene expression. CircRNAs can act as miRNA sponges, reducing miRNA-mediated repression of other targets, a mechanism implicated in various pathophysiological processes, including oncogenesis. Methods: We propose a mathematical model describing the dynamics of miRNA–mRNA–protein interactions, extending existing frameworks for miRNA–mRNA regulation. A qualitative analysis of the associated nonlinear differential equations system is performed. Results: We prove the boundedness of all positive solutions, establish the existence of a unique positive attracting equilibrium, and provide a mathematical perspective on the crosstalk mechanism in protein production. Conclusions: The effectiveness of ceRNA interactions depends on the relative abundance of miRNAs and their targets. This highlights the ongoing debate regarding the biological impact of low-abundance RNA transcripts on miRNA-mediated regulation.

## 1. Introduction

Protein production is homeostatically regulated through a precise balance of synthesis and degradation, maintaining correct intracellular concentrations. This fine-tuning is essential for cellular function, and its dysregulation is associated with malignancy, chemoresistance and sepsis.

Protein synthesis is regulated by multiple layers, including epigenetic mechanisms, with microRNAs playing a critical role.

MicroRNAs regulate gene expression post-transcriptionally, are evolutionarily conserved, and are generated via a defined biogenesis pathway involving Drosha, Dicer, and the RNA-induced silencing complex (RISC). Initially, non-coding RNA transcripts were dismissed as “junk DNA,” but the seminal work of Calin and Croce [1] demonstrated that microRNAs are actively involved in cancer pathogenesis. Today, their roles in tumorigenesis and chemotherapy resistance are well established [2].

The canonical function of microRNAs is the inhibition of protein synthesis at the post-transcriptional level; however, they also possess numerous non-canonical roles, including interactions with other non-coding RNAs [3].

Understanding microRNAs within the broader network of non-coding RNAs is therefore essential: each microRNA targets multiple mRNAs, and each mRNA is regulated by multiple microRNAs. A comprehensive understanding of these interactions is crucial for elucidating the dynamics of protein production.

As previously shown, microRNAs are negative regulators of gene expression, but in turn mRNAs also function as negative regulators of microRNA activity. This function is achieved through a competitive mechanism involving two or more mRNAs that are all inhibited by the same microRNA. This mechanism has been called competing endogenous RNAs (ceRNAs), and it can be understood as a true “language of communication” between numerous microRNAs and mRNAs. This so-called “ceRNA hypothesis” allows the analysis of regulatory action through a complex network of microRNAs and mRNAs that influence each other, thus ensuring fine-tuning functions of gene expression. In short, the expression of a gene (the production of a certain protein) can be influenced not only by the inhibitory action of one or more microRNAs, but also by the effect of a second mRNA that competes for the same microRNA. The function of this second mRNA has been described as a “sponge” (it acts as if it “absorbs” the shared microRNA).

This theory has regained attention with the strong demonstration of the sponge function of circular RNAs in various pathological mechanisms, including cancer. A potential use of circular RNAs as sponges represents an extremely promising therapeutic perspective. The ceRNA hypothesis appears logical and appealing for this reason.

However, it must be said that the hypothesis is still highly controversial. The main objection (Denzler et al. [4]) is that they cannot act as “sponges” to titrate miRNAs away from other normal targets except under conditions where the abundance of microRNAs and their targets is equivalent. Some authors believe that “The ceRNA hypothesis is controversial because it is difficult to imagine how the change in expression of individual miRNA targets, which each typically contribute a minuscule fraction of the target abundance, could possibly influence enough miRNA molecules to affect regulation of other targets.” (Denzler et al. [4]).

In this context, our study employs an ODE-based modeling approach to investigate the functional links among microRNAs, messenger RNAs (*m*RNAs), and protein synthesis, having as a starting point the model of mRNA—microRNA dynamics formulated by Figliuzzi et al. [5]. To be more specific, the proposed mathematical model describes the interaction between one microRNA (denoted by μ) and two messenger RNAs ($m_{1}$ and $m_{2}$, respectively), and, in addition to [6,7], it also incorporates the dynamics of both complexes and protein production. The resulting effects on the production of the corresponding proteins (denoted by $p_{1}$ and $p_{2}$) were also investigated.

Consider the following ODE system model

(1) $$\begin{array}{ccc} \frac{dm_{i}}{dt} & = & b_{i}-d_{i}m_{i}-k_{i}^{+}m_{i}\mu +k_{i}^{-}c_{i},\;\;i=1,2\\ \frac{d\mu }{dt} & = & \beta -\delta \mu -\left(k_{1}^{+}m_{1}+k_{2}^{+}m_{2}\right)\mu +\\ & + & \left(k_{1}^{-}+\kappa_{1}\right)c_{1}+\left(k_{2}^{-}+\kappa_{2}\right)c_{2}\\ \frac{dc_{i}}{dt} & = & -\left(\sigma_{i}+k_{i}^{-}+\kappa_{i}\right)c_{i}+k_{i}^{+}m_{i}\mu ,\;\;i=1,2\\ \frac{dp_{i}}{dt} & = & -\gamma_{i}p_{i}+\alpha_{i}m_{i},\;\;i=1,2 \end{array}$$

Here $m_{1}$ and $m_{2}$ are the concentrations of the two species of *m*RNA, μ is the concentration of the microRNA, $c_{1}$ and $c_{2}$ are the concentrations of the complexes, and $p_{1}$ and $p_{2}$ represent the protein concentrations, associated to $m_{1}$ and $m_{2}$, respectively. The coefficients $b_{i}$, i=1,2 and β are the transcription rates of the *m*RNAs and the microRNA, respectively. Further, $d_{i}$, δ, $\sigma_{i}$ and $\gamma_{i}$ are the degradation rates of the *m*RNAs, microRNA, complexes and proteins, while $k_{i}^{+}$, $k_{i}^{-}$, $\kappa_{i}$ and $\alpha_{i}$, i=1,2, are kinetic constants associated with the mass action rates of reactions. All constants are positive.

The ODE system (1) is a typical open enzymatic reaction model. The *m*RNA—microRNA dynamics have been studied in a series of previous papers ([5,6,8,9,10]).

We conduct a qualitative study of this model, including existence, uniqueness and boundedness of the solutions, but focused on the stability and nature of equilibrium points and obtained biologically relevant results such as return to equilibrium and protein dynamics under competition. In particular, we highlight the phenomenon of different competing species of messenger RNA (targeting the same microRNA) and the way in which this competition influences the protein production. These qualitative findings are consistent with experimental observations, particularly regarding stability and the effects of competition on protein dynamics. This suggests that the proposed model provides a reasonable approximation of real, though highly complex, biological phenomena.

We demonstrate that the system has a single positive equilibrium point, and that this point is asymptotically (globally) stable. In practical terms, this means that if the system is affected by some perturbations, it will naturally return to its equilibrium state without requiring any external correction.

Our model also considers the competition between $m_{1}$ and $m_{2}$, focusing on how this competition influences protein concentrations. To study this, we compared two scenarios:

(A) One mRNA, $m_{1}$.
(B) Two mRNA, $m_{1}$ and $m_{2}$.

We prove that, at equilibrium, $m_{1}$ and $p_{1}$ in scenario (B) are significantly higher than in scenario (A). This occurs because the microRNA μ acts as an inhibitor of protein production: when another messenger $m_{2}$ is present, it competes with $m_{1}$ for binding to μ, which reduces the inhibitory effect on $m_{1}$ and allows more protein to be produced. More broadly, our findings suggest that under certain biological conditions—such as appropriate rates of degradation and translation—a microRNA network can reach a single, stable state. In this state, the system reliably returns to equilibrium after perturbations, reflecting the robustness observed in many real biological processes [6,10,11].

The proposed model should be interpreted as a minimal deterministic framework rather than as a complete representation of endogenous ceRNA networks. The main goal of our research is to study (confirm or reject) the ceRNA hypothesis, starting from a deterministic ODE mathematical model generally accepted in the literature and without extending the discussion to a wider range of models, various biological assumptions or parameter estimation from in vivo biological data.

## 2. Mathematical Results

Positive solutions.

We are interested only in the study of positive solutions of system (1), obtaining the following result (see Appendix A):

**Theorem** **1.** *All positive solutions of the system (1) are bounded, hence they can be extended on $\left[t_{0},\infty \right)$ for every initial moment $t_{0}\geq 0$.*

Equilibria.

We are now searching the positive equilibrium points of system (1). The following result holds—proof in Appendix B.

**Theorem** **2.** *For every set of positive parameters $b_{i},\beta ,d_{i},\delta ,\sigma_{i}$, $k_{i}^{+},k_{i}^{-},\kappa_{i},\alpha_{i}$, the system (1) has a unique equilibrium point $\left(\tilde{m_{i}},\tilde{\mu },{\tilde{c}}_{i},\tilde{p_{i}}\right)$ in $R_{+}^{7}$ (i=1,2). Furthermore*

$$\tilde{m_{i}}\in \left(0\;,\;\frac{b_{i}}{d_{i}}\right),\;\;\;\tilde{\mu }\in \left(0\;,\;\frac{\beta }{\delta }\right),\;\;\;\tilde{c_{i}}\in \left(0\;,\;\frac{b_{i}\beta \;k_{i}^{+}}{d_{i}\delta \;\bar{\sigma_{i}}}\right),\;\;\;\tilde{p_{i}}\in \left(0\;,\;\frac{\alpha_{i}\;b_{i}}{\gamma_{i}\;d_{i}}\right).$$

Stability.

**Figure 1:**
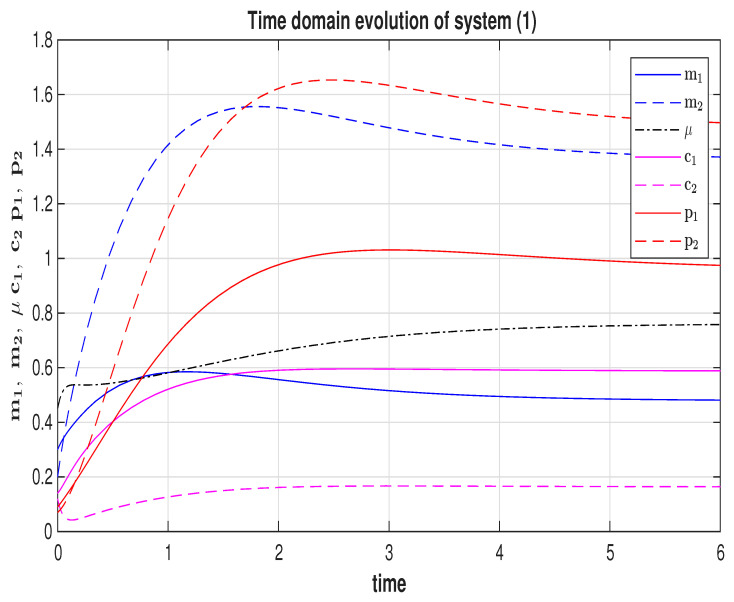
A typical time-domain evolution of system (1). One can notice the asymptotic stability (proved in Appendix C).

It is well known that, for this type of enzymatic reactions, the quasi steady-state assumption, $\frac{dc_{i}}{dt}=0$, i=1,2, applies. Various simulations (see Figure 1) suggest that the complexes $c_{i}$ are exhibiting a visible shorter settling time than the other five variables—see also Remark 7 in [12]. Under this assumption, we prove the following result (see Appendix C):

**Theorem** **3.** *For every positive set of parameters $b_{i},\beta$, $d_{i},\delta ,\sigma_{i}$, $k_{i}^{+},k_{i}^{-},\kappa_{i}$, $\gamma_{i},\alpha_{i}$ the equilibrium $\left(\tilde{m_{i}},\tilde{\mu },\tilde{p_{i}}\right)$, i=1,2 is asymptotically stable. Furthermore, the equilibrium $\left(\tilde{m_{i}},\tilde{\mu },\tilde{p_{i}}\right)$, i=1,2 is a global attractor in the positive orthant $R_{+}^{5}$ for the system (A9).*

It is worthwhile to mention that, from a technical point of view, the global attractor stability result does not depend on the quasi steady-state assumption: asymptotic stability can be proved, appealing to a more involved mathematical technique (see [12]). In other words, this assumption does not make a qualitative difference in the statement of the main results of the paper, it only keeps the mathematical complexity at a lower level.

## 3. ceRNA Hypothesis (Cross-Talking)

In this section we analyze how the equilibria concentration values of the mRNAs and proteins interact with each other in several scenarios (ceRNA). Toward this goal, let us consider the following cases:

(i) One mRNA $m_{1}$ and one protein $p_{1}$.
(ii) Two mRNAs ($m_{1}$ and $m_{2}$) and two proteins ($p_{1}$, and $p_{2}$), respectively.

To be more specific, we investigate how the equilibrium values of $m_{1}$ and $p_{1}$ (from case (i)) change in the presence of a second mRNA, $m_{2}$ (case (ii)). This dependence is usually called cross-talking (see [5]). The corresponding ODE model of case (i) is given by

(2) $$\begin{array}{ccc} \frac{dm_{1}}{dt} & = & b_{1}-d_{1}m_{1}-k_{1}^{+}m_{1}\mu +k_{1}^{-}c_{1}\\ \frac{d\mu }{dt} & = & \beta -\delta \mu -k_{1}^{+}m_{1}\mu +\left(k_{1}^{-}+\kappa_{1}\right)c_{1}\\ \frac{dc_{1}}{dt} & = & -\left(\sigma_{1}+k_{1}^{-}+\kappa_{1}\right)c_{1}+k_{1}^{+}m_{1}\mu \\ \frac{dp_{1}}{dt} & = & -\gamma_{1}p_{1}+\alpha_{1}m_{1}, \end{array}$$

and, according to Theorem 2, it has a unique equilibrium point $\left({\tilde{m}}_{1}^{0},{\tilde{\mu }}^{0},{\tilde{c}}_{1}^{0},{\tilde{p}}_{1}^{0}\right)$ in $R_{+}^{4}$, with

$${\tilde{m}}_{1}^{0}\in \left(0\;,\;\frac{b_{1}}{d_{1}}\right),\;\;{\tilde{\mu }}^{0}\in \left(0\;,\;\frac{\beta }{\delta }\right),\;\;{\tilde{c_{1}}}^{0}\in \left(0\;,\;\frac{b_{1}\beta \;k_{1}^{+}}{d_{1}\delta \;\bar{\sigma_{1}}}\right),\;\;{\tilde{p}}_{1}^{0}\in \left(0\;,\;\frac{\alpha_{1}\;b_{1}}{\gamma_{i}\;d_{1}}\right).$$

We analyze how ${\tilde{m}}_{1}^{0}$ and ${\tilde{p}}_{1}^{0}$ are changing in the presence of a second *m*RNA, $m_{2}$, targeted by the same microRNA μ—as in system (1). From Equation (A7) we get

$$\frac{b_{1}-d_{1}m_{1}}{A_{1}m_{1}}=\frac{b_{2}-d_{2}m_{2}}{A_{2}m_{2}},$$

hence

(3) $$\tilde{m_{1}}=\frac{b_{1}A_{2}\;m_{2}}{b_{2}A_{1}+\left(d_{1}A_{2}-d_{2}A_{1}\right)\;m_{2}}.$$

By differentiating $\tilde{m_{1}}$ with respect to $m_{2}$ in (3) we obtain

$$\frac{d\tilde{m_{1}}}{dm_{2}}=\frac{b_{1}b_{2}\;A_{1}A_{2}}{{\left(b_{2}A_{1}+\left(d_{1}A_{2}-d_{2}A_{1}\right)\;m_{2}\right)}^{2}}>0.$$

Consequently

(4) $$\frac{d\tilde{p_{1}}}{dm_{2}}=\frac{d\tilde{p_{1}}}{dm_{1}}\;\frac{dm_{1}}{dm_{2}}>0.$$

**Figure 2:**
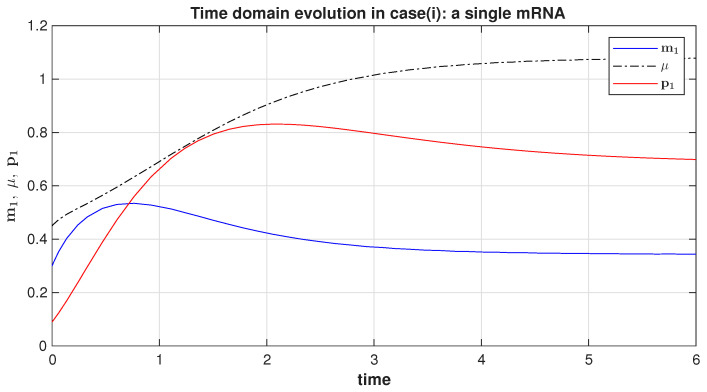
A typical time evolution of system (2): a single mRNA.

**Figure 3:**
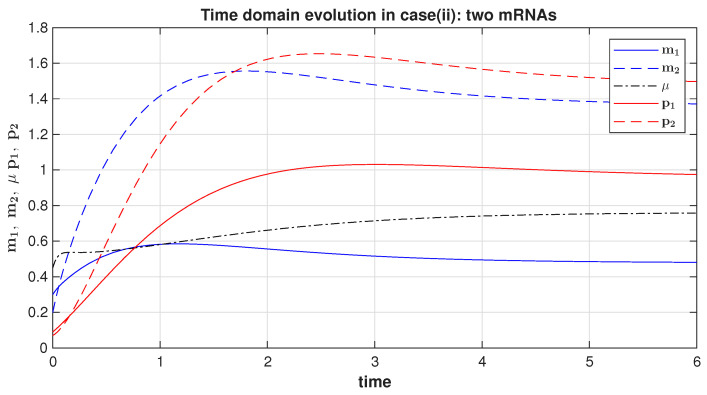
In the presence of a second mRNA (denoted by $m_{2}$), the equilibrium value of $p_{1}$ is increasing compared with case (i); see Figure 2.

Thus, we have proved the following result (see also Figure 2 and Figure 3).

**Theorem** **4.** *In the presence of $m_{2}$, the value of $m_{1}$ at equilibrium is increasing. Consequently, the value of $p_{1}$ at equilibrium is also increasing in the presence of $m_{2}$.*

Theorem 4 provides a theoretical basis for the emergence of ceRNA-like competitive behavior under the assumptions of the proposed model. Therefore, the result should be interpreted as supporting the mathematical plausibility of ceRNA-like crosstalk, rather than as biological validation of the ceRNA hypothesis.

Furthermore, relying on various illustrative numerical simulations, we analyzed to what extent the interaction proved in Theorem 4 is biologically relevant (for quantitative experimental results see [4,13]). To this goal, we studied three scenarios

**Figure 4:**
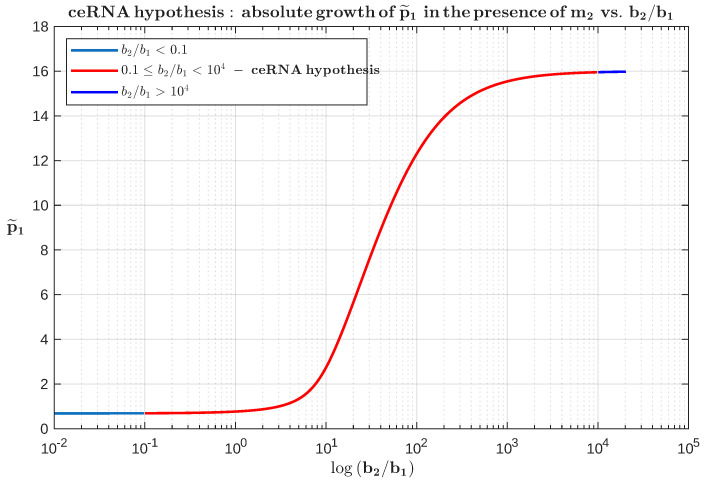
Equilibrium level of protein $p_{1}$ as a function of the relative transcription rate $b_{2}/b_{1}$. The figure shows the equilibrium value ${\tilde{p}}_{1}$, corresponding to $m_{1}$ plotted against $\log(b_{2}/b_{1})$, where $b_{1}$ and $b_{2}$ are the transcription rates of $m_{1}$ and $m_{2}$, respectively. The simulation illustrates that when $b_{2}\ll b_{1}$, the equilibrium level of $p_{1}$ remains approximately unchanged. A substantial increase in ${\tilde{p}}_{1}$ is observed only when $b_{1}$ and $b_{2}$ are of comparable magnitude, indicating a parameter region compatible with ceRNA-like competition. When $b_{2}\gg b_{1}$, the equilibrium level of $p_{1}$ again reaches an approximately constant regime. Thus, the predicted ceRNA-like effect is restricted to an intermediate stoichiometric window rather than occurring across the entire range of transcription-rate ratios.

**Figure 5:**
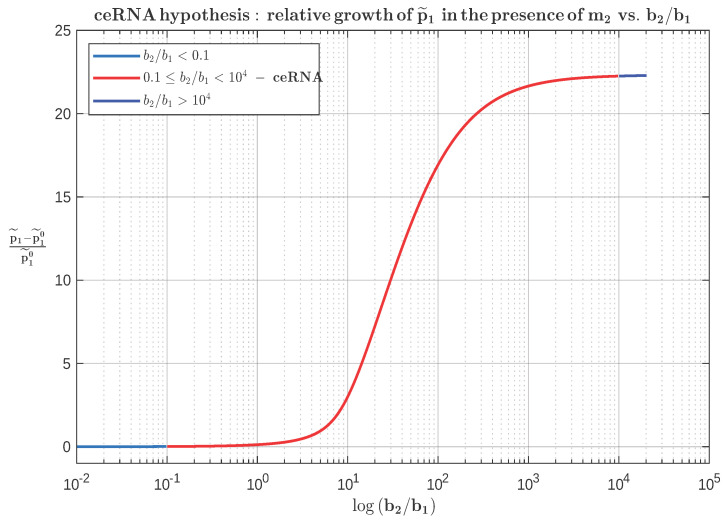
Relative increase in the equilibrium level of protein $p_{1}$ induced by the presence of a second mRNA target $m_{2}$. The quantity $\left({\tilde{p}}_{1}-{\tilde{p}}_{1}^{0}\right)/{\tilde{p}}_{1}^{0}$ is plotted as a function of $\log(b_{2}/b_{1}))$, where ${\tilde{p}}_{1}^{0}$ denotes the equilibrium level of $p_{1}$ in the single-target case involving only $m_{1}$, and ${\tilde{p}}_{1}$ denotes the equilibrium level of $p_{1}$ in the two-target case involving both $m_{1}$ and $m_{2}$. The figure indicates that the presence of the second mRNA produces a biologically relevant relative increase in $p_{1}$ only when the transcription rates $b_{1}$ and $b_{2}$ are comparable. For highly unbalanced transcription rates, either $b_{2}\ll b_{1}$ or $b_{2}\gg b_{1}$, the relative increase is limited, supporting the conclusion that ceRNA-like cross-talk occurs only within a restricted stoichiometric regime.

**Figure 6:**
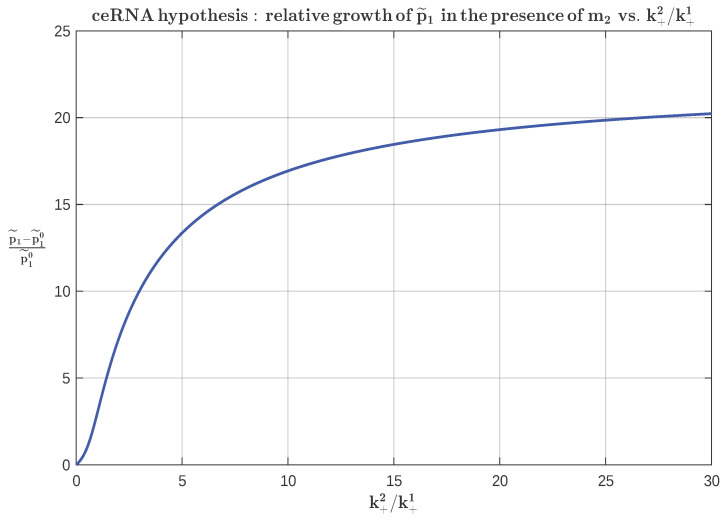
Relative growth of ${\tilde{p}}_{1}$ versus $k_{2}^{+}/k_{1}^{+}$. Here, instead of the variation of the production rates $b_{1}$ and $b_{2}$, we consider the variations of the reaction rates $k_{1}^{+}$ and $k_{2}^{+}$, respectively. Similar conclusions to those in Figure 5 can be drawn.

(a) The mRNAs $m_{2}$ and $m_{1}$ have similar production rates and kinetic constants associated with the corresponding mass reaction rates (see Figure 2 and Figure 3). The presence of a second mRNA $m_{2}$ leads to an increase of the equilibrium value of the produced protein, ${\tilde{p}}_{1}$, as one can see in the corresponding lines 2 and 3 in Table 1 and in column 2 in Table 2. Thus, under these parameter conditions, the model predicts the emergence of ceRNA-like competitive behavior—see also the red zone of the graphics in Figure 4 and Figure 5, respectively.
(b) If the mRNA $m_{2}$ has substantially lower production rate than $m_{1}$, i.e., $b_{2}<<b_{1}$—see, for instance, the light-blue zone on the graphic in the Figure 4 and Figure 5, as well as the equilibrium values in the corresponding lines 2 and 4 in Table 1 and in column 3 in Table 2—then the equilibrium value of the produced protein, ${\tilde{p}}_{1}$, remains actually quite close to the equilibrium value of the produced protein ${\tilde{p}}_{1}^{0}$, determined by the presence of only one mRNA. This behavior is also reproduced due to the obvious symmetry between $m_{1}$ and $m_{2}$: if $m_{2}$ has a substantially greater production rate than $m_{1}$, i.e., $b_{2}>>b_{1}$, then again ${\tilde{p}}_{1}$ changes slightly compared to ${\tilde{p}}_{1}^{0}$—see the dark-blue zone on the graphic in the Figure 4 and Figure 5, as well as the equilibrium values in column 3 in Table 2. Consequently, the ceRNA hypothesis is likely to be not biologically relevant in this case.
(c) A similar analysis can be done when one investigates the effect of the variation of kinetic constants $k_{1}^{+}$ and $k_{2}^{+}$ on the equilibrium values of the protein production. Thus, if the interaction of $m_{2}$ with μ is much lower than the interaction of $m_{1}$ with μ, i.e., $k_{2}^{+}<<k_{1}^{+}$ (or, much greater, i.e., $k_{2}^{+}>>k_{1}^{+}$ ), then ${\tilde{p}}_{1}$ does not change too much in comparison to ${\tilde{p}}_{1}^{0}$—see Figure 6, as well as the equilibrium values in the corresponding lines 2 and 5 in Table 1. We get similar conclusions with situation (b), that is, likely no biological relevance of the ceRNA assumption.

We summarize the previous observations, looking at the absolute and relative variations of ${\tilde{p}}_{1}$ with respect to the magnitude of $\log\frac{b_{2}}{b_{1}}$—see Figure 4 and Figure 5, respectively. These theoretical conclusions are in line with [4,13]; additional details are given in Section 4.

## 4. Discussion

MicroRNAs (miRNAs) play a pivotal role in the post-transcriptional regulation of gene expression by mediating translational repression and promoting mRNA degradation, thereby modulating intracellular protein levels. Disruptions in the tightly regulated processes of protein synthesis and folding are critically implicated in the pathogenesis of numerous severe disorders, including malignant neoplasms and neurodegenerative diseases such as Alzheimer’s disease. A mathematical mRNA–microRNA–protein ODE model provides a mechanistic framework for integrating experimentally validated interactions and quantitatively analyzing gene expression regulation. In this study, we focus on miRNA-mediated protein synthesis through mRNA–miRNA interactions, particularly within the competing endogenous RNA (ceRNA) framework, where target RNAs compete for shared miRNAs and thereby regulate miRNA activity. Building on previous theoretical models of ceRNA interactions [14,15,16], our ODE model highlights the essential role of miRNAs in molecular stability and protein homeostasis, offering insight into mechanisms relevant to pathophysiology and potential therapeutic strategies. Our findings are consistent with Le Chatelier’s principle [17], which states that a system at equilibrium responds to perturbations by shifting in a direction that counteracts the disturbance. This principle is conceptually analogous to biological homeostasis, whereby organisms maintain stable internal conditions through adaptive responses to internal and external changes. The model shows that regardless of initial conditions, solutions converge to a unique positive and stable equilibrium for protein synthesis homeostasis. However, this analogy should not be interpreted as a mechanistic equivalence with Le Chatelier’s principle or with the full complexity of biological homeostasis. It also describes cross-talk between two mRNAs competing for a shared microRNA, thereby illustrating a possible mechanism underlying stable ceRNA interactions [18]. However, this interpretation should not be considered mechanistically equivalent to Le Chatelier’s principle or to the full complexity of biological homeostasis, and further experimental validation is required. Because the analysis is mainly equilibrium-based, the model captures long-term behavior but does not fully capture transient dynamics, stochastic molecular fluctuations, or noise-driven effects, especially at low transcript copy numbers. Future extensions should include transient-response analysis, stochastic simulations, and larger regulatory networks involving multiple mRNAs and miRNAs. Additional biological mechanisms, such as miRNA recycling, target-site accessibility, cooperative binding, AGO/RISC saturation, subcellular localization, feedback regulation, and heterogeneous RNA turnover, should also be incorporated to better reflect in vivo heterogeneity.

Our analysis suggests that the biological relevance of the ceRNA hypothesis is constrained by stringent numerical conditions. A key limitation is the large number of parameters, which may generate various numerical solutions, while preserving the model’s qualitative properties. Thus, the results should be interpreted within biological reductionism, if all parameters remain constant.

Numerical simulations were performed under controlled parameter conditions using MATLAB R2025b routines, specifically ode23 and ode45. These simulations were intended as qualitative parameter explorations rather than as probabilistic Monte Carlo analyses or experimentally calibrated predictions. Parameter values are illustrative [5] and not calibrated to specific experimental datasets; therefore, numerical results should be viewed as qualitative explorations rather than quantitative biological predictions. Biologically grounded parameterization would require experimental estimates of miRNA–mRNA binding affinities, kinetic constants, degradation rates, translation rates, miRNA recycling efficiency, and transcript copy numbers. Given the number of kinetic and degradation parameters, future work should include local and global sensitivity analyses to identify the most strongly influencing parameters in the ceRNA-like behavior. Nevertheless, the qualitative results remain valid for all positive parameter values.

Circular RNAs (circRNAs) have gained attention as potential miRNA sponges and important components of ceRNA regulation. Their structure provides enhanced stability and longer half-lives compared with linear RNAs, while circRNAs containing multiple miRNA-binding sites may compete with target mRNAs for shared miRNAs, thereby modulating miRNA availability (Liu and Chen [19]; Salmena et al. [20]; Poliseno et al. [21]). These features suggest that circRNAs may satisfy key requirements for effective ceRNA-mediated regulation. From a theoretical perspective, circRNAs may act as efficient molecular sponges due to their structural stability and resistance to exonucleolytic degradation [22]. This property also supports their potential therapeutic and diagnostic relevance, including their use as engineered miRNA sponges and stable biomarkers in diseases such as cancer. However, the endogenous sponge activity of circRNAs remains debated. Although circRNAs may exert ceRNA effects in specific biological contexts, many endogenous circRNAs may be insufficiently abundant to induce broad miRNA derepression. Therefore, physiologically meaningful effects are likely limited to high-expression or high-affinity cases, consistent with current discussions on the quantitative requirements for ceRNA activity—see [23,24].

**Figure 7:**
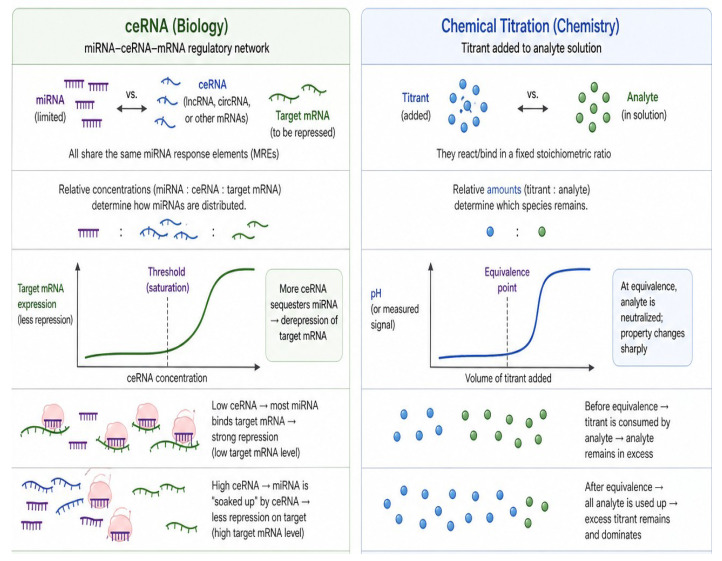
ceRNA Hypothesis vs. Chemical Titration. A Clear Analogy. ceRNAs act as molecular sponges for shared miRNAs. When ceRNA exceeds a threshold, miRNAs are sequestered, relieving repression on target mRNAs. Titration is based on stoichiometric binding. At the equivalence point, the system switches behavior because the limiting reagent is exhausted.

Additionally, targeting ceRNA networks through circRNA or miRNA modulation may enable precise and combinatorial therapeutic strategies. However, clinical translation requires overcoming major challenges, including efficient delivery, context-dependent effects, and off-target interactions. A key prerequisite for therapeutic ceRNA modulation is that the competitive mechanism must be both chemically feasible and biologically relevant, a central issue addressed by the present model. Our findings show that relative molecular abundance, particularly copy-number ratios within the ceRNA network, is a critical determinant of system behavior. Protein output is modulated only within a narrow concentration range, indicating a restricted operational window for effective ceRNA crosstalk. The relationship shown in Figure 7 resembles a classical chemical titration curve, suggesting that ceRNA interactions may follow an underlying quantitative logic analogous to established physicochemical systems.

**Figure 8:**
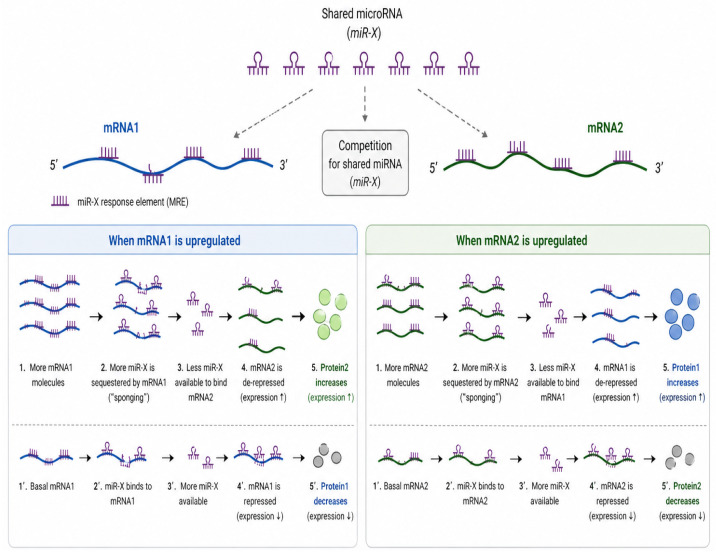
ceRNA Hypothesis. Competition between mRNA1 and mRNA2. mRNA1 and mRNA2 compete for the same pool of miRNAs. Increasing one RNA relieves repression on the other, leading to increased mRNA and protein expression.

In both titration and ceRNA, outcomes depend on competition for a limiting component. This mechanism is illustrated in Figure 8, where mRNA1 and mRNA2 compete for the same miRNA pool: upregulation of one mRNA sequesters miRNAs and reduces their availability to bind the other mRNA, thereby relieving repression and increasing protein expression.In titration, the analyte and titrant react according to defined molar ratios, whereas in ceRNA networks multiple RNA species compete for a limited pool of miRNAs. Thus, miRNAs may be regarded as limiting regulatory molecules, while ceRNAs act as competing species that sequester them. When ceRNA abundance crosses a critical threshold, miRNAs become effectively sequestered, leading to target mRNA derepression and increased protein output. This resembles the equivalence point in titration, where a sharp change in a pH measurable property occurs. Accordingly, both systems display nonlinear, threshold-like behavior governed by stoichiometric relationships. However, this analogy should not be overextended. Unlike classical titration, ceRNA interactions are dynamic, reversible, probabilistic, and strongly context-dependent, involving multiple molecular species and regulatory mechanisms, rather than a single fixed reaction pair. In our simulations, we examined whether the ODE-based model can identify the concentration range in which ceRNA crosstalk becomes biologically meaningful. Specifically, we tested the hypothesis proposed by Denzler and colleagues [13], that miRNA-mediated repression is relieved in a threshold-like manner only at high target-site abundance, and that changes in miRNA target abundance are otherwise unlikely to substantially affect gene expression or metabolism through a ceRNA mechanism. Our results suggest that, although ceRNA effects may theoretically occur across diverse concentration regimes, their biologically relevant impact is confined to a narrow range of copy-number ratios. In practice, such conditions are unlikely to occur under typical physiological or pathophysiological settings. In conclusion, our findings demonstrate that the ceRNA hypothesis is theoretically plausible within the framework of the proposed model. However, the biological relevance of ceRNA-mediated crosstalk remains an open question, particularly given the restrictive numerical conditions required for its manifestation under physiological circumstances. Our results indicate that ceRNA-like competition may emerge mathematically only within restricted parameter regimes, particularly when miRNA and target abundances are comparable. Therefore, the model supports theoretical plausibility of ceRNA-like interactions under defined stoichiometric conditions, but it does not constitute biological validation of the ceRNA hypothesis. As suggested by Denzler [4,13] and Bosson [25], such effects may occur predominantly in specific experimental or pathophysiological contexts. The maximal influence of one mRNA on the expression of another is expected when miRNA and target transcript concentrations are comparable (Hausser and Zavolan [16]; Denzler [13]). Future work should extend this framework by incorporating stochastic effects, transient dynamics, experimentally grounded parameterization, sensitivity analysis, and larger regulatory networks.

## Figures and Tables

**Table 1:** Coefficient and equilibrium values corresponding to Figure 1, Figure 2, Figure 3, Figure 4 and Figure 5, respectively.

Figure	Coefficient Values	Equilibrium Values
Figure 1	$b_{1}=2$, $b_{2}=2.7$, β=1.5, $d_{1}=0.25$,	$\tilde{m_{1}}=0.4785$, $\tilde{m_{2}}=1.3616$,
	$d_{2}=0.3$, δ=1, $k_{1}^{+}=10$, $k_{2}^{+}=3$,	$\tilde{\mu }=0.7613$,
	$k_{1}^{-}=3$, $k_{2}^{-}=5$, $\kappa_{1}=2.5$, $\kappa_{2}=12$,	$\tilde{c_{1}}=0.5876$, $\tilde{c_{2}}=0.1637$,
	$\sigma_{1}=0.7$, $\sigma_{2}=2$, $\alpha_{1}=2$, $\alpha_{2}=2.5$,	$\tilde{p_{1}}=0.9571$, $\tilde{p_{2}}=1.4800$
	$\gamma_{1}=1$, $\gamma_{2}=2.3$	
Figure 2	$b_{1}=2$, $b_{2}=0$, β=1.5, $d_{1}=0.25$,	${\tilde{m}}_{1}^{0}=0.3430$,
	$d_{2}=0$, δ=1, $k_{1}^{+}=10$, $k_{2}^{+}=0$,	${\tilde{\mu }}^{0}=1.0813$,
	$k_{1}^{-}=3$, $k_{2}^{-}=0$, $\kappa_{1}=2.5$, $\kappa_{2}=0$,	${\tilde{p}}_{1}^{0}=0.6860$
	$\sigma_{1}=0.7$, $\sigma_{2}=0$, $\alpha_{1}=2$, $\alpha_{2}=0$,	
	$\gamma_{1}=1$, $\gamma_{2}=0$	
Figure 3	$b_{1}=2$, $b_{2}=2.7$, β=1.5, $d_{1}=0.25$,	$\tilde{m_{1}}=0.4785$, $\tilde{m_{2}}=1.3616$,
	$d_{2}=0.3$, δ=1, $k_{1}^{+}=10$, $k_{2}^{+}=3$,	$\tilde{\mu }=0.7613$,
	$k_{1}^{-}=3$, $k_{2}^{-}=5$, $\kappa_{1}=2.5$, $\kappa_{2}=12$,	$\tilde{p_{1}}=0.9571$, $\tilde{p_{2}}=1.4800$
	$\sigma_{1}=0.7$, $\sigma_{2}=2$, $\alpha_{1}=2$, $\alpha_{2}=2.5$,	
	$\gamma_{1}=1$, $\gamma_{2}=2.3$	
Figure 4	$b_{1}=2$, $b_{2}=0.1$, β=1.5, $d_{1}=0.25$,	$\tilde{m_{1}}=0.3468$, $\tilde{m_{2}}=0.0376$,
	$d_{2}=0.3$, δ=1, $k_{1}^{+}=10$, $k_{2}^{+}=8.75$,	$\tilde{\mu }=1.0688$,
	$k_{1}^{-}=3$, $k_{2}^{-}=5$, $\kappa_{1}=2.5$, $\kappa_{2}=12$,	$\tilde{p_{1}}=0.6937$, $\tilde{p_{2}}=0.0408$
	$\sigma_{1}=0.7$, $\sigma_{2}=2$, $\alpha_{1}=2$, $\alpha_{2}=2.5$,	
	$\gamma_{1}=1$, $\gamma_{2}=2.3$	
Figure 5	$b_{1}=2$, $b_{2}=2.7$, β=1.5, $d_{1}=0.25$,	$\tilde{m_{1}}=0.3674$, $\tilde{m_{2}}=7.2166$,
	$d_{2}=0.3$, δ=1, $k_{1}^{+}=10$, $k_{2}^{+}=0.1$,	$\tilde{\mu }=1.0062$,
	$k_{1}^{-}=3$, $k_{2}^{-}=5$, $\kappa_{1}=2.5$, $\kappa_{2}=12$,	$\tilde{p_{1}}=0.7349$, $\tilde{p_{2}}=7.8441$
	$\sigma_{1}=0.7$, $\sigma_{2}=2$, $\alpha_{1}=2$, $\alpha_{2}=2.5$,	
	$\gamma_{1}=1$, $\gamma_{2}=2.3$	

**Table 2:** Significant values of $\frac{b_{2}}{b_{1}}$, ${\tilde{p}}_{1}$ and $\frac{{\tilde{p}}_{1}}{{\tilde{p}}_{1}^{0}}-1$.

$b_{2}/b_{1}$	1/50	1	50
$\tilde{p_{1}}$ (Figure 4)	0.6901	0.9571	13.1038
$\frac{{\tilde{p}}_{1}}{{\tilde{p}}_{1}^{0}}-1$ (Figure 5)	0.0060	0.3951	18.1010

## Data Availability

The data supporting the findings of this study are available from the corresponding author upon reasonable request.

## Appendix A. Study of the Positive Solutions

Define $X^{T}:=\left[\begin{array}{ccccccc} m_{i} & m_{2} & \mu & c_{1} & c_{2} & p_{1} & p_{2} \end{array}\right]$. Then, the differential equations system (1) can be rewritten as

$$\frac{dX}{dt}=F\left(X\right),$$

where *F* is the appropriate vector field defined on $R^{7}$ and associated to the system (1). For every $X_{0}=\left(m_{i}^{0},\mu^{0},c_{i}^{0},p_{i}^{0}\right)\in R^{7}$, i=1,2, denote by $X(t;t_{0},X_{0})$ the solution of the Cauchy problem

$$\frac{dX}{dt}=F\left(X\right),\;\;X\left(t_{0}\right)=X_{0}.$$

**Remark** **A1.**
*Since F is a polynomial vector field, the Existence and Uniqueness Theorem applies. Furthermore, one can show that the positive orthant $R_{+}^{7}$ is a (positively) invariant set for the system (1)—as has been proceeded in [12].*

**Remark** **A2.**
*Provided that $m_{i}$, i=1,2, are bounded functions (as it will be shown in Appendix B), the last two equations in (1) have the following solutions*

$$p_{i}\left(t\right)=p_{i}\left(t_{0}\right)\;e^{-\gamma_{i}\left(t-t_{0}\right)}+\alpha_{i}\;e^{-\gamma_{i}t}\;\int_{t_{0}}^{t}m_{i}\left(\tau \right)\;e^{\gamma_{i}\tau }d\tau .$$

We are interested only in the study of positive solutions of system (1)—see also Remark A1. Let us first prove that the solutions are bounded.

**Theorem** **A1.**
*All positive solutions of system (1) are bounded, hence they can be extended on $\left[t_{0},\infty \right)$ for every initial moment $t_{0}\geq 0$.*

**Proof.** We shall use the same technique as in [12], based on [26] and on Section 4, Chapter 3, in [27]. By adding the first and the fourth equations in system (1) we get

$$\frac{dm_{1}}{dt}+\frac{dc_{1}}{dt}=-d_{1}m_{1}-\left(\sigma_{1}+\kappa_{1}\right)c_{1}+b_{1}\leq -\rho_{1}\left(m_{1}+c_{1}\right)+b_{1},$$

where $\rho_{1}=\min\left\{d_{1},\sigma_{1}+\kappa_{1}\right\}$. It results that $m_{1}+c_{1}$ is upper bounded. Analogously, $m_{2}+c_{2}$ is upper bounded as well. Add now the third, the fourth and the fifth equation in (1); it then follows that

$$\frac{d\mu }{dt}+\frac{dc_{1}}{dt}+\frac{dc_{2}}{dt}=-\delta \mu -\sigma_{1}c_{2}-\sigma_{2}c_{2}+\beta \leq -\omega \left(\mu +c_{1}+c_{2}\right),$$

where $\omega =\min\{\delta ,\sigma_{1},\sigma_{2}\}$. Hence $\mu +c_{1}+c_{2}$ is upper bounded. Since $m_{i}$, μ, $c_{i}$, i=1,2, are all positive, it results that they are all upper bounded. Consequently, the positive solutions of (1) can be extended on $\left[t_{0},\infty \right)$ for every initial moment $t_{0}\geq 0$; see Figure 1. □

## Appendix B. Equilibria

We are now searching the positive equilibrium points of system (1). The following result holds.

**Theorem** **A2.**
*For every set of positive parameters $b_{i},\beta$, $d_{i},\delta ,\sigma_{i}$, $k_{i}^{+},k_{i}^{-},\kappa_{i},\alpha_{i}$, system (1) has a unique equilibrium point $\left(\tilde{m_{i}},\tilde{\mu },{\tilde{c}}_{i},\tilde{p_{i}}\right)$ in $R_{+}^{7}$ (i=1,2). Furthermore*

$$\tilde{m_{i}}\in \left(0\;,\;\frac{b_{i}}{d_{i}}\right),\;\;\;\tilde{\mu }\in \left(0\;,\;\frac{\beta }{\delta }\right),\;\;\;\tilde{c_{i}}\in \left(0\;,\;\frac{b_{i}\beta \;k_{i}^{+}}{d_{i}\delta \;\bar{\sigma_{i}}}\right),\;\;\;\tilde{p_{i}}\in \left(0\;,\;\frac{\alpha_{i}\;b_{i}}{\gamma_{i}\;d_{i}}\right).$$

**Proof.** These equilibrium points are the solutions of the following algebraic system of equations (i=1,2):

(A1) $$\begin{array}{ccc} 0 & = & b_{i}-d_{i}m_{i}-k_{i}^{+}m_{i}\mu +k_{i}^{-}c_{i} \end{array}$$

(A2) $$\begin{array}{ccc} 0 & = & \beta -\delta \mu -\left(k_{1}^{+}m_{1}+k_{2}^{+}m_{2}\right)\mu +\\ & + & \left(k_{1}^{-}+\kappa_{1}\right)c_{1}+\left(k_{2}^{-}+\kappa_{2}\right)c_{2} \end{array}$$

(A3) $$\begin{array}{ccc} 0 & = & -\left(\sigma_{i}+k_{i}^{-}+\kappa_{i}\right)c_{i}+k_{i}^{+}m_{i}\mu \end{array}$$

(A4) $$\begin{array}{ccc} 0 & = & -\gamma_{i}p_{i}+\alpha_{i}m_{i},\;\; \end{array}$$

Let

(A5) $$\begin{array}{cc} \bar{\sigma_{i}}:=\sigma_{i}+k_{i}^{-}+\kappa_{i},\;\;A_{i}:=\frac{\left(\sigma_{i}+\kappa_{i}\right)k_{i}^{+}}{\bar{\sigma_{i}}},\;\;B_{i}:=\frac{\sigma_{i}k_{i}^{+}}{\bar{\sigma_{i}}} & \\ & \mathrm{where}\;\;A_{i}>B_{i},\;\;i=1,2. \end{array}$$

It follows immediately from Equation (A3) that

(A6) $$c_{i}=\frac{B_{i}}{\sigma_{i}}\;m_{i}\mu ,\;\;i=1,2.$$

Replace now $c_{i}$ in (A1) and obtain

(A7) $$m_{i}=\frac{b_{i}}{d_{i}+A_{i}\mu }\in \left(0\;,\;\frac{b_{i}}{d_{i}}\right),\;i=1,2.$$

Introduce now the above expressions of $m_{i}$ and $c_{i}$ in (A2) and get

$$\mu =\frac{\beta }{\delta +B_{1}m_{1}+B_{2}m_{2}}\in \left(0\;,\;\frac{\beta }{\delta }\right),$$

or, equivalently,

(A8) $$\mu =\frac{\beta }{\delta +\frac{B_{1}b_{1}}{d_{1}+A_{1}\mu }+\frac{B_{2}b_{2}}{d_{2}+A_{2}\mu }}.$$

Let $f:R_{+}\to R_{+}$ be defined by

$$f\left(x\right)=\frac{\beta }{\delta +\frac{B_{1}b_{1}}{d_{1}+A_{1}x}+\frac{B_{2}b_{2}}{d_{2}+A_{2}x}}.$$

Obviously f(0)>0 and $f\left(x\right)\leq \frac{\beta }{\delta }$ for all positive *x*. It follows that the equation f(x)=x has a unique solution $\tilde{\mu }$ in $\left(0\;,\;\frac{\beta }{\delta }\right)$. Thus, the system has a unique equilibrium point $\left(\tilde{m_{i}},\tilde{\mu },{\tilde{c}}_{i},\tilde{p_{i}}\right)$, with $\tilde{m_{i}}$ given by Equation (A7), $\tilde{c_{i}}$ by Equation (A6) and

$$\tilde{p_{i}}=\frac{\alpha_{i}}{\gamma_{i}}\;\tilde{m_{i}}\in \left(0\;,\;\frac{\alpha_{i}\;b_{i}}{\gamma_{i}\;d_{i}}\right),\;i=1,2.$$

□

**Remark** **A3.**
*From the proof of Theorem 2 one can see that*

$$\begin{array}{cc} \tilde{m_{i}}\in \left(\frac{B_{i}}{d_{i}+A_{i}\frac{\beta }{\delta }}\;,\;\frac{b_{i}}{d_{i}}\right),\;\;\tilde{\mu }\in \left(\frac{\beta }{\delta +B_{1}\frac{b_{1}}{d_{1}}+B_{2}\frac{b_{2}}{d_{2}}}\;,\;\frac{\beta }{\delta }\right), & \\ \tilde{p_{i}}\in & \left(\frac{\alpha_{i}}{\gamma_{i}}\;\frac{B_{i}}{d_{i}+A_{i}\frac{\beta }{\delta }}\;,\;\frac{\alpha_{i}}{\gamma_{i}}\;\frac{b_{i}}{d_{i}}\right),\;i=1,2. \end{array}$$

*Moreover,*

$$\lim_{t\to \infty }p_{i}\left(t\right)=\tilde{p_{i}},\;i=1,2,$$

*where $p_{i}\left(t\right)$ have been introduced in Remark A2.*

## Appendix C. Stability

It is well known that, for this type of enzymatic reaction, the quasi steady-state assumption, $\frac{dc_{i}}{dt}=0$, i=1,2, applies [28,29]. Various simulations (see Figure 1) suggest that the complexes $c_{i}$ are exhibiting a visible shorter settling time than the other five variables—see also Remark 7 in [12]. Under this assumption, system (1) becomes:

(A9) $$\begin{array}{ccc} \frac{dm_{i}}{dt} & = & b_{i}-d_{i}m_{i}-A_{i}m_{i}\mu ,\;i=1,2\\ \frac{d\mu }{dt} & = & \beta -\delta \mu -(B_{1}m_{1}+B_{2}m_{2})\mu \\ \frac{dp_{i}}{dt} & = & -\gamma_{i}p_{i}+\alpha_{i}m_{i},\;i=1,2 \end{array}$$

where

$$A_{i}=\frac{\left(\sigma_{i}+\kappa_{i}\right)k_{i}^{+}}{\bar{\sigma_{i}}},\;\;\;B_{i}=\frac{\sigma_{i}k_{i}^{+}}{\bar{\sigma_{i}}},\;\;A_{i}>B_{i}.$$

Then, the Jacobian matrix associated to system (A9) is given by:

(A10) $$J\left(m_{1},m_{2},\mu ,p_{1},p_{2}\right)=\left[\begin{array}{ccccc} -D_{1} & 0 & -A_{1}m_{1} & 0 & 0\\ 0 & -D_{2} & -A_{2}m_{2} & 0 & 0\\ -B_{1}\mu & -B_{2}\mu & -\Delta & 0 & 0\\ \alpha_{1} & 0 & 0 & -\gamma_{1} & 0\\ 0 & \alpha_{2} & 0 & 0 & -\gamma_{2} \end{array}\right]$$

where $D_{i}=d_{i}+A_{i}\mu$, i=1,2, and $\Delta =\delta +B_{1}m_{1}+B_{2}m_{2}$, respectively. Straightforward computations yield its characteristic polynomial:

$$P\left(\lambda \right)=\left(\lambda +\gamma_{1}\right)\left(\lambda +\gamma_{2}\right)\;Q\left(\lambda \right),$$

where

$$\begin{array}{c} Q\left(\lambda \right)=\lambda^{3}+\left(B_{1}m_{1}+B_{2}m_{2}+A_{1}\mu +A_{2}\mu +d_{1}+d_{2}\right)\lambda^{2}+\\ \;(A_{2}B_{1}m_{1}\mu +A_{1}B_{2}m_{2}\mu +A_{1}A_{2}\mu^{2}+d_{1}B_{1}m_{1}+d_{2}B_{1}m_{1}+\\ \;d_{1}B_{2}m_{2}+d_{2}B_{2}m_{2}+d_{1}A_{2}\mu +d_{2}A_{1}\mu +d_{1}d_{2})\lambda +\\ \;(d_{1}A_{2}B_{1}m_{1}\mu +d_{2}A_{1}B_{2}m_{2}\mu +d_{1}d_{2}B_{1}m_{1}+d_{1}d_{2}B_{2}m_{2}). \end{array}$$

The third degree polynomial Q(λ) has all coefficients positive and is trivially verifying the Hurwitz test. Hence P(λ) is Hurwitz as well. Thus the Jacobian matrix is stable at any point $\left(m_{i},\mu ,p_{i}\right)\in R_{+}^{5}$, i=1,2. Accordingly, the next Theorem holds.

**Theorem** **A3.**
*For every positive set of parameters $b_{i},\beta$, $d_{i},\delta ,\sigma_{i}$, $k_{i}^{+},k_{i}^{-},\kappa_{i}$, $\gamma_{i},\alpha_{i}$ the equilibrium $\left(\tilde{m_{i}},\tilde{\mu },\tilde{p_{i}}\right)$, i=1,2 is asymptotically stable. Furthermore, the equilibrium $\left(\tilde{m_{i}},\tilde{\mu },\tilde{p_{i}}\right)$, i=1,2 is a global attractor in the positive orthant $R_{+}^{5}$ for the system (A9).*

**Proof.** Recall (see Theorem 1) that all positive solutions of (A9) are also bounded and can be also extended on $\left[t_{0},\infty \right)$ for every initial moment $t_{0}\geq 0$. Then Corollary 3.4.8 in [30] can be applied and we deduce that there exists a global attractor in $R_{+}^{5}$ which is connected. But we have just proved in Proposition 3 that $\left(\tilde{m_{i}},\tilde{\mu },\tilde{p_{i}}\right)$, i=1,2, is the unique (hence isolated) asymptotically stable equilibrium point of the system (A9). Hence, it results that this equilibrium is the global attractor in $R_{+}^{5}$. □
